# Economic Evaluation of PRIMROSE—A Trial-Based Analysis of an Early Childhood Intervention to Prevent Obesity

**DOI:** 10.3389/fendo.2018.00104

**Published:** 2018-03-14

**Authors:** Nora Döring, Niklas Zethraeus, Per Tynelius, Jeroen de Munter, Diana Sonntag, Finn Rasmussen

**Affiliations:** ^1^Prevention, Intervention and Mechanisms in Public Health, Department of Public Health Sciences, Karolinska Institutet, Stockholm, Sweden; ^2^Department of Learning, Informatics, Management and Ethics, Karolinska Institutet, Stockholm, Sweden; ^3^Centre for Epidemiology and Community Medicine, Stockholm County Council, Stockholm, Sweden; ^4^Medical Faculty of Heidelberg University, Mannheim Institute of Public Health, Social Prevention and Medicine (MIPH), Mannheim, Germany; ^5^Department of Health Science, University of York, York, United Kingdom; ^6^Department of Health Sciences, Lund University, Lund, Sweden

**Keywords:** economic evaluation, childhood, obesity, prevention, RCT

## Abstract

**Background:**

Childhood obesity is a major clinical and economic health concern. Alongside the clinical understanding of obesity, there is a growing interest in designing and implementing interventions that are worth their money given the scarce resources in the health care sector. This study is one of the first efforts to provide evidence by assessing the effects and costs of a population-based primary prevention intervention targeting pre-school children attending child health centers in Sweden.

**Methods:**

The economic evaluation is based on the PRIMROSE cluster-randomized controlled trial aiming to establish healthy eating and physical activity among pre-school children (9–48 months of age) through motivational interviewing applied by trained nurses at child health centers. The cost-effectiveness is assessed over the trial period from a societal perspective. The primary outcome was BMI at age 4. Cost data was prospectively collected alongside the trial. Scenario analyses were carried out to identify uncertainty.

**Results:**

The estimated additional mean total costs of the PRIMROSE intervention were 342 Euro (95% CI: 334; 348) per child. During pre-school years direct costs mainly consist of training costs and costs for the additional time used by nurses to implement the intervention compared to usual care. Early indirect costs mainly consist of parents’ absence from work due to their participation in the intervention. The incremental cost-effectiveness ratio in the base case analysis was 3,109 Euro per 1 BMI unit prevented.

**Conclusion:**

We cannot provide evidence that the PRIMROSE intervention is cost-effective, given the uncertainty in the effect measure. Until further evidence is provided, we recommend resources to be spent elsewhere within the field of obesity prevention. Furthermore, to achieve valid and reliable cost-effectiveness results, the economic evaluation of obesity prevention programs in early childhood should incorporate the life time impact to capture all relevant costs and benefits.

## Introduction

Despite signs of stabilization (1, 2), the burden of childhood overweight is still considerable in many westernized countries. Severe health concerns for the individual (3), but also significant societal and economic consequences (4, 5) have raised awareness among policy makers and researchers likewise to address childhood obesity through primary prevention strategies already at early ages. Still, the evidence base around the prevention of childhood obesity is far from conclusive (6–10). Nevertheless, primary health care providers and (pre-) school settings may be encouraged to address and implement behavioral counseling and other interventions as long as they do not cause harm. Yet, given the scarce resources of most health care systems, decision makers need to prioritize and shed light on opportunity costs. So far, little is known about the costs of universal population-based primary childhood obesity prevention interventions, especially in the European settings (11). Health economic evaluations are often neglected when designing and conducting intervention studies. The lack of relevant, individual, and prospectively collected data hamper the meaningful conduction of cost-effectiveness analyses. In a recent systematic review, only six studies addressing the cost-effectiveness of obesity prevention programs in early childhood were identified, and only three of them were based on a randomized trial (11) This paper aims to critically assess the costs and evaluate the economic benefits of a population-based primary prevention intervention embedded in regular child health services targeting first time parents and their children.

## Materials and Methods

### Study Design and Setting

This paper describes an economic evaluation of the PRIMROSE cluster-randomized primary prevention trial, where the costs and outcomes of the intervention were compared with those of usual care from a societal perspective. A societal perspective implies that costs also outside the health care system were included in addition to direct health care costs, i.e., productivity losses, due to participation in the trial. The present economic evaluation is an analysis of the costs and health effects of the PRIMROSE trial during the intervention period only, i.e., up to age 4.

### The PRIMROSE Cluster-Randomized Controlled Trial

The PRIMROSE trial evaluated the effectiveness of an early childhood obesity intervention delivered in the first 4 years of life, embedded in regular child health services in Sweden. Details of the study design and the intervention components have been reported previously (12, 13). In brief, the study included 1,355 families with 1,369 infants. The intervention took place at child health care centers (CHCs), which were randomized into interventions (*n* = 31) and control units (*n* = 28). The intervention consisted of nine sessions in a time frame of approximately 39 months delivered by specially trained nurses. The intervention aimed to assist first time parents in promoting healthy food and physical activity habits in their children and in changing their own health behaviors if needed through the application of motivational interviewing (MI). The intervention was targeting eating pattern (i.e., regular meals together with the family, no force feeding/eating), food choices (i.e., consumption of fruit and vegetables, reduced consumption of soft drinks and snacks), and physical activity (i.e., incorporating physical activity in the everyday routine, reducing sedentary time). The intervention components were mainly targeted at the parents to become role models for their children and to increase parental self-efficacy for behavioral change. Prior to intervention, nurses attended a 5-day workshop, including an introduction to healthy nutrition, physical activity, learning theory, and social cognitive theory (SCT), as well as training in MI. During the course of the intervention, nurses received extensive and tailored feedback on their MI performance (14). Ethical approval (2006/525-31/2) was obtained from the Regionala Etikprövningsnämnden Stockholm (The Ethical Review Board Stockholm). All parents of the participating children gave informed written consent.

### Comparator

Families in the control CHCs were only offered the regular age-related health check-ups of Swedish child health services, which focused on physical development and immunizations and less attention is paid to children’s health behavior (15). Swedish CHCs are free or charge and attended by nearly all families in Sweden.

### Measurement of Clinical Outcomes

Children’s weight, height, and waist circumferences were objectively measured by study nurses at each visit to the CHC. The primary outcome was BMI at age four, applying the IOTF references for defining cut-offs (16). Secondary outcomes were mother’s objectively measured anthropometrics as well as children’s and mother’s physical activity and food habits (13). For the current analysis, only the primary outcome, i.e., BMI at age 4, was considered.

### Measurement of Costs

Costs of the intervention program included costs of a 5-day workshop offered to intervention nurses, costs of MI training, and supervision of nurses and costs of implementation. The costs of the workshop were obtained from collected invoices and salary contracts. We collected prospectively data on costs to deliver the intervention, including staff’s time to deliver the intervention and parents’ time to take part. This information was then supplemented with parents’ average net salaries to estimate productivity losses due to participation in the intervention, based on the human capital approach. In line with current guidelines (17), we excluded the costs for research and development and any costs associated with evaluation or administration of the trial. Costs are indexed to the year 2015 and displayed in Euro using the average exchange rate from 2015 (1 Euro = 9.3 SEK).

### Statistical Analysis and Uncertainty Analysis

We compared the total costs of the PRIMROSE intervention to the costs of usual care. Costs and effects were derived from participant-level data. The incremental cost-effectiveness ratio (ICER) was expressed as cost per 1 BMI unit prevented. The method of non-parametric bootstrapping was applied using EXCEL, where 1,000 costs and outcome pairs were generated (with replacement) (18). The results were illustrated by using cost-effectiveness acceptability curves, in which the probability that the PRIMROSE intervention is cost-effective was illustrated for different theoretical willingness-to-pay (WTP) levels for prevention of 1 BMI unit.

### Scenario Analysis

We conducted two types of scenario analysis for calculating the intervention costs. First, intervention costs were calculated based on a per-protocol basis. Instead of individual uptake and duration of meetings, we assumed full uptake (seven face-to-face meetings and two telephone meetings) and the duration of meetings according to the manual specification as previously reported (13). Missing information on parents’ attendance was imputed based on the observed distribution of parents’ attendances during respective meetings. In a second scenario analysis, we halved the observed duration of meetings. This is to partly account for potential overlap with usual health care during the intervention meetings, but also to allow for a shorter duration of intervention meetings if implemented in the current CHC practices. The effect measure was kept constant.

### Decision-Making Beyond Cost-Effectiveness

In addition to the quantitative assessment of the cost-effectiveness, we applied the criteria developed by the ACE-Obesity Working Group, which are intended to incorporate other, broader aspects of decision-making. The criteria included were “strength of evidence,” “equity,” “feasibility of implementation,” “acceptability of stakeholders,” “sustainability,” and “side-effects” (19).

## Results

At follow-up, there were 1,148 children with data on weight and height at age 4. Intervention and control children at follow-up were very similar with regards to demographic characteristics and baseline characteristics (12).

### Main Intervention Effect

The main results of the trial have been published elsewhere (12). In brief, there was no statistical significant indication for improvement in the primary outcome measure of children’s BMI at age 4. While the intervention effect pointed in the “right” direction, the estimate was too small to reach statistical significance with respect to group differences in the children’s BMI at age 4 [β = −0.11, 95% confidence interval (CI): −0.31 to 0.08] (12).

### Total Costs

The estimated mean total costs per participant in the intervention group were 453 Euro (Min = 177, Max = 740) in comparison to 111 Euro (Min = 0, Max = 246) in the control group for the usual care. The mean additional costs for carrying out this interventions were 342 Euro (95% CI: 334; 348) per participant. The main costs components of the education program were costs of the workshop, costs of MI training and supervision, (Table 1) and costs of implementation of the intervention program (Table 2). The largest component of PRIMROSE costs arose from delivery of the intervention within the CHC settings. The large intervention costs variation is mainly driven by meeting uptake.

**Table 1:** Summary of unit cost information, data sources, and assumptions for education and training of nurses.

Type of cost	Description	Unit costs	Source	Assumptions
**Education**				

**Personnel time**				

Nurse	10 nurses per education, 40 h	17.9 Euro/h	Statistics Sweden, salary statistics Primrose database	Average wage rate
Nutritionist	Food habits and physical activity training, 3 h	21.5 Euro/h	Statistics Sweden, salary statistics Primrose database	Average wage rate
Psychologist	SCT, learning theory, and some CBT training, 3 h	23.9 Euro/h	Statistics Sweden, salary statistics Primrose database	Average wage rate
MI trainer (psychologist)	28 h	23.9 Euro/h	Statistics Sweden, salary statistics Primrose database	Average wage rate
Instructor	2 instructors, 16 h	23.9 Euro/h	Statistics Sweden, salary statistics Primrose database	Average wage rate
Supervisor (MINT)	5 supervisors, 8 h	19.9 Euro/h	Statistics Sweden, salary statistics Primrose database	Average wage rate
Project coordinator	40 h	16.6 Euro/h	Primrose database	

**Other costs**				

Catering and materials	Includes coffee/tea, lunches, and snacks	3,023 Euro	Primrose database	Calculation based on invoices made for one education session
Travel	Two-way train ride	55 Euro	Estimate	No information on mode of transportation; Average costs for medium distance train ride

	Per nurse			1,441.4 Euro
	Per child			110.8 Euro

**Training**				

**Personnel time**				

Nurse	9 occasions, 30 min feedback on the telephone	17.9 Euro/h	Statistics Sweden, salary statistics Primrose database	Average wage rate, full maintenance

Supervisor (MINT)	9 occasions, 1 h preparation, 30 min on the telephone	19.9 Euro/h	Statistics Sweden, salary statistics	Average wage rate

**Other costs**				

Coding of MI conversations	6 codings	55 Euro	Invoice	

Recording device	One recording device per nurse	22 Euro	Invoice	

	Per nurse			867.9 Euro
	Per child			66.6 Euro

*SCT, social cognitive theory; CBT, cognitive behavioral therapy; MI, motivational interviewing; MINT, motivational interviewing network of trainers*.

**Table 2:** Summary of unit cost information, data sources, and assumptions for intervention delivery.

Type of cost	Description	Unit costs	Source	Assumptions
**Group meeting**				

Number of group meetings	On average five parental units per group meeting with both parents being present	1	Primrose manual	
Length of meeting^a^		1 h and 23 min	Primrose database	
Nurse		17.9 Euro/h	Statistics Sweden, salary statistics Primrose database	Average wage rate
Parents^b^				
Father (7.7%) Mother (53.2%)		18.5 Euro/h 14.3 Euro/h	Statistics Sweden, salary statistics, estimate	Average wage rate

**Other costs**				

Travel	Two-way ride	5 Euro	Estimate	Average collective communication cost for short distance

**Individual meeting**				

Number of individual meetings		6	Primrose manual	
Length of meeting^a^		53 min	Primrose database	
Nurse		17.9 Euro/h	Statistics Sweden, salary statistics Primrose database	Average wage rate
Parents^b^				
Father (7.8%) Mother (54.1%)		18.5 Euro/h 14.3 Euro/h	Statistics Sweden, salary statistics, estimate	Average wage rate

**Telephone meeting**				

Number of telephone meetings		2	Primrose manual	
Length of meeting^a^		22 min	Primrose database	
Nurse		17.9 Euro/h	Statistics Sweden, salary statistics Primrose database	Average wage rate
Parents^b^				
Father (8.9%) Mother (77%)		18.5 Euro/h 14.3 Euro/h	Statistics Sweden, salary statistics, estimate	Average wage rate

*^a^Mean length reported in the trial*.

*^b^Percentage refers to parental presence during the meetings, otherwise both parents were present*.

### Cost of Education

In total, 67 nurses received the PRIMROSE education, which involved a 5-day workshop, including an introduction to nutrition, physical activity, learning theory, and SCT, as well as training in MI. The MI training part of the workshop consisted of 3.5 days, with 8 h of training per day. Seven workshops were conducted, each with an average of 10 participating nurses. The workshops were led by a senior clinical psychologist with extensive experience in leading MI workshops, and membership of the Motivational Interviewing Network of Trainers. On average, two more licensed clinical psychologists assisted as workshop instructors. On the last day, participants’ supervisor’s joined the workshop. In total, there were 10 supervisors, which were each responsible for on average five nurses. On average five supervisors participated per workshop (20).

### Costs of Further MI Training and Supervision

After the workshop intervention, nurses were offered feedback on their MI performance at nine occasions (four training sessions before the PRIMROSE intervention and five sessions with children in the PRIMROSE intervention group). 35 of 51 nurses (69%) completed all nine supervision sessions that were planned to last for 30 min and to be based on at least 20 min of audio-recorded session time with parents of young children. In total, six sessions (one training session and five intervention sessions) were coded for quality of MI performance by the motivational interviewing treatment integrity-code (14).

### Costs of Implementation

The PRIMROSE intervention consisted of one group meeting, six individual meetings and two telephone meetings. These meetings were conducted as add-on of the usual care provided at the CHC. In Table 2, the mean duration of visits is reported. On average, 54% of all CHC visits were done by mothers only. If both parents were present, the meetings were on average 15 min longer. To calculate the costs of implementation, we used individual trial data concerning uptake of meetings, parental presence, and duration of meetings.

**Figure 1:**
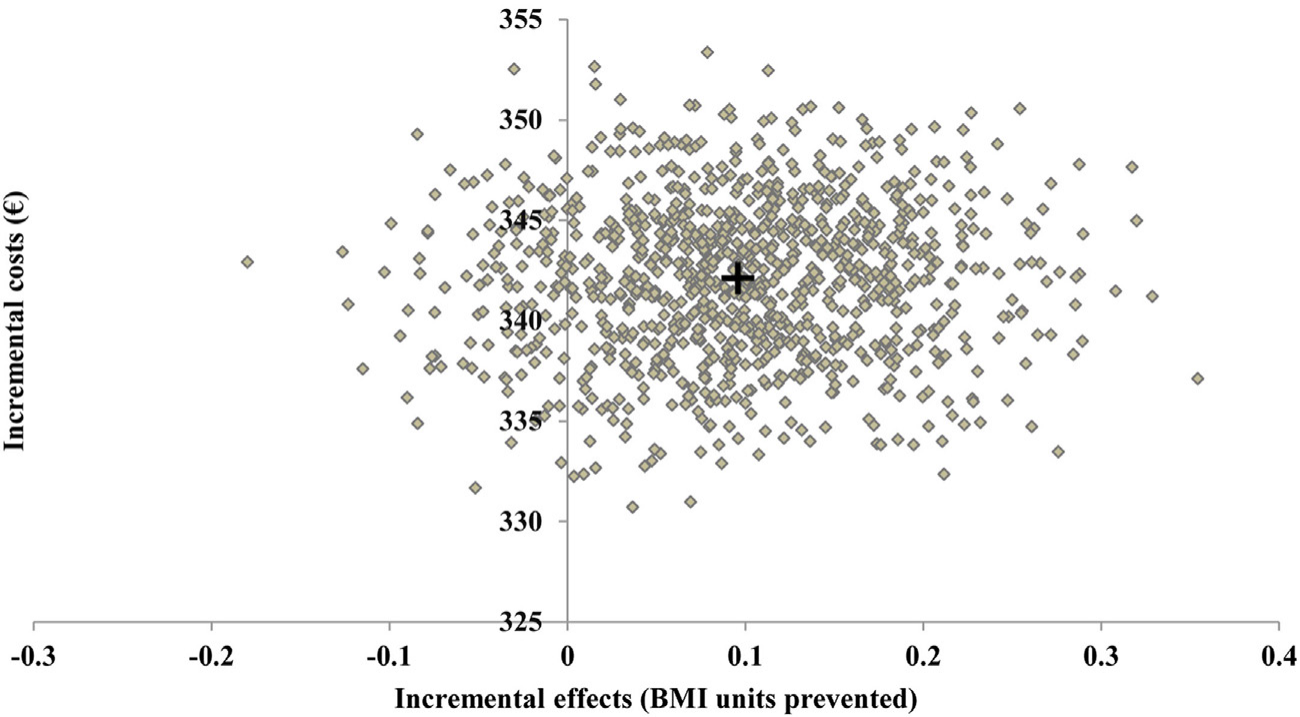
Incremental costs and incremental effects of the PRIMROSE intervention on the cost-effectiveness plane. Results of 1,000 Monte Carlo simulations. + = point estimate.

In the RCT, the point estimate of the ICER was 3,109 Euro per 1 BMI unit prevented. The bootstrapped estimates of incremental costs and incremental benefits (represented by BMI units prevented) of the PRIMROSE interventions are presented in the cost-effectiveness plane (Figure 1). About 11% of the bootstrapped pairs were dominated, meaning the PRIMROSE intervention costs more for less effect. Yet, the vast majority of the bootstrapped ICER estimates indicate increased benefits and greater costs.

**Figure 2:**
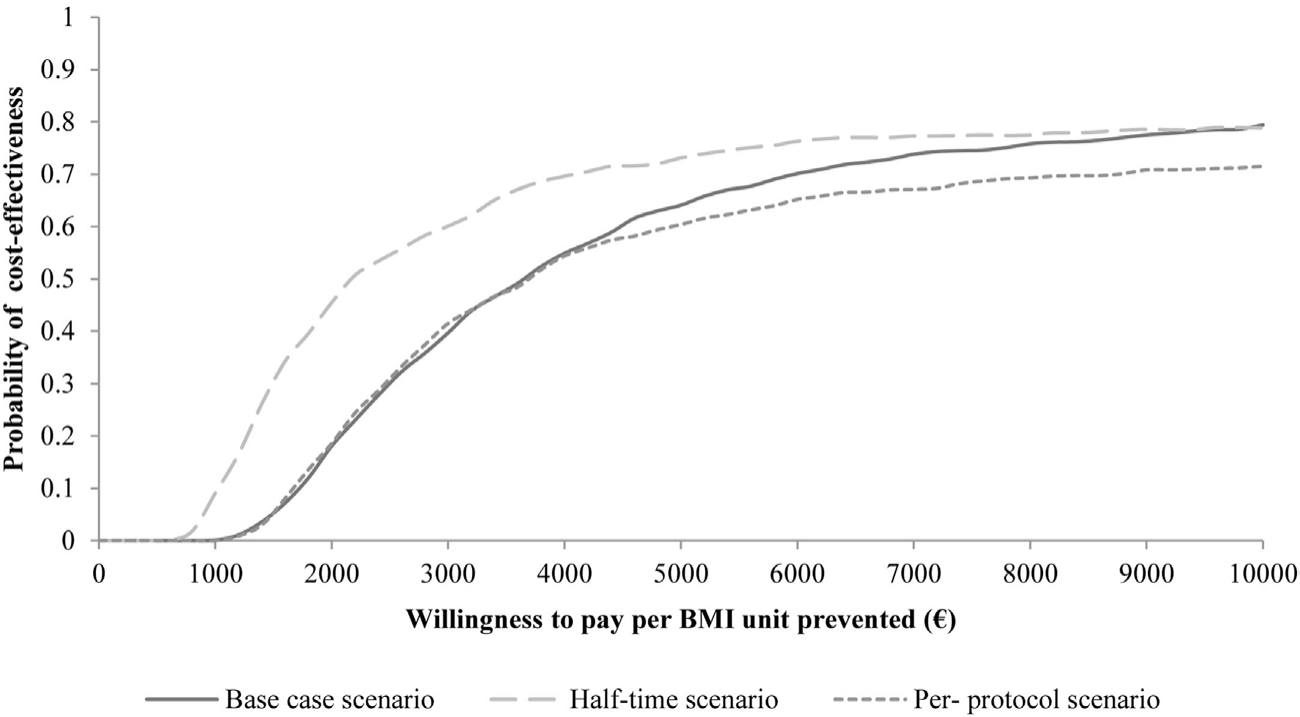
Cost-effectiveness acceptability curves of PRIMROSE for three scenarios “Base case scenario,” “Half-time Scenario,” and “Per-protocol Scenario.”

Regarding the scenario analyses, the per-protocol analysis resulted only in marginal differences in intervention costs with a corresponding ICER of 3,553 Euro per BMI unit. When we assumed the meeting time to be halved, the ICER was reduced to 2,128 Euro per BMI unit. The cost-effectiveness acceptability curve presents the probability of cost-effectiveness of the PRIMROSE intervention given different levels of WTP per avoidance of 1 BMI unit for all three scenarios (Figure 2). It shows that by halving the meeting times the probability of cost-effectiveness can be increased by approximately 20%. With increasing WTP, one can observe an approximation of probabilities of cost-effectiveness for all three scenarios.

### Decision-Making Beyond Cost-Effectiveness

When looking beyond the cost-effectiveness, key concerns relevant for decision-making were around the strength of evidence, feasibility, and sustainability (Table 3). While the intervention effect pointed in the right direction, the group difference was not statistically significant. The evidence was limited and additional, possible larger, RCTs might be needed to confirm the results. Furthermore, more research is needed to reflect on the clinical relevance of small effects on BMI during early childhood. The additional time needed by health professionals cannot be disregarded and need to be carefully evaluated. In addition, there are some issues that require resolutions to ensure sustainability, including, among others, the need of ongoing training and supervision of nurses. When implemented in practice, it can be assumed that equity issues can be neglected given that nearly 100% of the Sweden living population attends the regular CHC meetings, irrespective of ethnicity or SES (21).

**Table 3:** Criteria for decision-making.

Strength of Evidence	Equity	Acceptability	Feasibility	Sustainability	Side-effects
– Large cluster-RCT – Results not statistically significant	– PRIMROSE population has higher SES compared to the general population – Targeted to Swedish speaking families only	– Positively received by nurses in the trial – No stigmatization (primary prevention) – On-top of regular child care	– Embedded in regular child health services – Potential problems concerning additional time required	– On-going training of nurses during the intervention, high quality manual – Possible issues: updating of manual, ensuring an adequate workforce of trained nurses, motivational interviewing competence	– Positive spill-over: potential impact on weight and health behavior on other family members – Possible unintended negative: potential feeling of lack of self-efficacy of parents, however, unlikely
Major concerns	Minor concerns	No concerns	Some concerns	Some concerns	No concerns

## Discussion

This is the first European trial-based economic evaluation of an early childhood obesity prevention intervention. While the intervention effect pointed in the favorable direction, there was no statistical significant BMI difference at age 4 between intervention and control groups. From a societal perspective, the incremental costs of the intervention were estimated to 342 Euro per participating family over 4 years. The corresponding ICER was 3,109 Euro per BMI unit prevented. As discussed elsewhere (12), the reasons for the non-significant effect size can be manifold and do not necessarily reflect an ineffective intervention. However, given the uncertainty combined with considerable opportunity costs, the current trial-based economic evaluation of PRIMROSE suggest that resources might be better used elsewhere within the field of obesity prevention.

The PRIMROSE intervention study and its economic evaluation has a number of strengths, including the large number of participants in the RCT and the prospective planning of the economic evaluation, which allowed the inclusion of detailed individual cost data. Combined with detailed measurements of individual participation time and national statistics on age adjusted mean salary information, we were able to also include individual productivity losses. However, we need to acknowledge that we had no accurate information on the individual employment situation (i.e., unemployment or parental leave), and, therefore, cannot exclude the possibility that parental productivity losses might be under- or overestimated. Given that societal costs often outweigh the direct health care costs, we recommend for future economic evaluation of RCTs a prospective and detailed collection of all relevant economic information. Furthermore, we did not have access to individual health care utilization data during the trial period, in addition to the healthcare provided by the CHCs. One may, however, assume that the vast amount of obesity related health care costs (and savings by prevention) occurs later in life, which was confirmed in the study of Hayes et al. showing only marginal differences in health care costs up to age 2 (22). However, over the subsequent 3 years, total health care costs of children with obesity were 1.62 (95% CI 1.12–2.36) times higher than among children with normal weight, which was driven by the higher risk of hospitalization (23). When comparing only children who were hospitalized, the differences were non-significant between the BMI groups. Therefore, more research on health care utilization during the early childhood is needed to also capture the possible short-term benefits of obesity prevention.

We are aware of only two other studies that conducted an economic evaluation that was restricted to the costs and effects during trial period for that age group (22, 24). The Australian trial-based economic evaluation of “Healthy Beginnings” reported an ICER of AUD 4,230 (≈2,950 Euro) per BMI unit prevented (22). Their intervention was conducted only over a period of 2 years, yet with a similar intensity of 8 home visits, in comparison to 7 meetings and 2 telephone meetings in the PRIMROSE intervention. The economic evaluation of the live, eat, and play (LEAP) intervention showed intervention costs close to AUD 5,000 borne by both the health care sector and the families (24). However, they also included costs borne by the family by changing the diet or physical activity habits. When excluding those, the costs over the 15 months trial period AUD 973, still more costly than PRIMROSE. The LEAP economic evaluation did not report any ICER, given their insignificant observed effect size. This is a similar case as in the PRIMROSE intervention, yet we followed the approach suggested by Drummond who argues for the importance of conducting economic evaluations even if the effect size does not reach statistical significance (17). This approach is also followed by Moodie et al. in their economic evaluation of the “Be active eat well intervention,” who calculated an ICER of AUD 547 (≈381 Euro) per BMI unit prevented during the trial period (25). Yet, the costs and effects were aggregated to the school level in a somewhat older population, which may hinder the direct comparison to our results.

Despite similar results to other studies, the judgment of whether such an intervention is cost-effective depends on the WTP of decision makers. Currently, there is no national or international threshold on WTP for the prevention of a BMI gain in childhood. Given the challenges of calculating QALYs for this age group (11, 26), we hope that our calculated ICER, similar to the one calculated by Hayes et al. (22) can serve as comparator for future economic evaluations, especially in the European setting. Next to the choice of outcome measure for economic evaluations during early childhood, the preferred choice of time horizon is also debatable. There are arguments to restrict economic evaluation of early childhood obesity prevention to the observation period. These include the lack of evidence on effect maintenance, the lack of evidence on the independent association of childhood obesity and adult onset of diseases, and the methodological challenges of linking childhood obesity to utility values. Furthermore, decision makers may also be interested in the immediate costs (or savings) of an intervention. At the same time, these calculations are likely to be very conservative and important costs and health parameters are missing that allow meaningful decision on resource allocation. To truly capture the cost-effectiveness of an intervention all consequences need to be considered. For preventive obesity intervention studies during early childhood this includes health consequences and societal costs over the entire life time. A model-based simulation study is a way of synthesizing the best available data on health effect and costs in the long-run also including consequences beyond the clinical trial period. In future economic evaluations of strategies for preventing obesity in early childhood, we recommend to combine clinical trial data with data from outside the trial using a modeling framework, taking into account the consequences on costs and benefits in the long-run.

## Conclusion

The economic evaluation of the PRIMROSE intervention demonstrated that even small intervention effects would be value for money under current modeling assumptions. However, given the uncertainty around the effect measure, resources might be better used elsewhere within the field of obesity prevention, until further evidence on effectiveness is provided. In addition, more research in the phases of design, implementation, evaluation, and maintenance of early childhood interventions for obesity is needed to provide policymakers and decision makers with the information they seek to allocate scarce resources in a more efficient and sustainable way. Furthermore, to achieve valid and reliable cost-effectiveness results, the economic evaluation of obesity prevention programs in early childhood should incorporate the life time impact to capture all relevant costs and benefits.

## Ethics Statement

The trial was approved by the Ethical Review Board in Stockholm, Sweden and was recorded in the ISRCTN registry (ISRCTN16991919). All subjects gave written informed consent in accordance with the Declaration of Helsinki.

## Author Contributions

ND substantially contributed to the design of the study, carried out the analysis, and drafted the initial manuscript. DS substantially contributed to the design of the study and the analysis, and provided substantial and critical feedback on the manuscript. JM and PT provided substantial feedback on the manuscript. NZ supported the data analysis and provided substantial feedback on the final manuscript. FR is the PI of the PRIMROSE intervention and contributed to the design of this study and provided substantial feedback on the final manuscript.

## Conflict of Interest Statement

The authors declare that the research was conducted in the absence of any commercial or financial relationships that could be construed as a potential conflict of interest.
